# A Novel Scale to Assess Parental Satisfaction of Dental Local Anesthetic Techniques in Children: A Cross-Sectional Study

**DOI:** 10.1155/2023/9973749

**Published:** 2023-05-19

**Authors:** Muaaz Alkhouli, Zuhair Al-Nerabieah, Mayssoon Dashash

**Affiliations:** Pediatric Dentistry, Faculty of Dentistry, Damascus University, Damascus, Syria

## Abstract

**Background:**

Pain control is one of the most important aspects that can affect parental satisfaction of the dental care provided for children. Dental local anesthesia has the highest impact on pain sensation of the children. However, there is no scale in the literature to assess parental satisfaction of dental local anesthetic techniques.

**Objectives:**

This study was aimed to assess the parental satisfaction with dental local anesthetic techniques for their children through designing a scale that reflects satisfaction and to study the validity and reliability of this scale.

**Methods:**

A cross-sectional observational study was conducted on 150 parents (102 mothers and 48 fathers). Two techniques of local anesthesia were used for each child participated in this study (inferior alveolar nerve block and computerized intraosseous anesthesia). The developed scale consisted of 20 items in a 5-point Likert scale. Half of the items were written in a negative format. Internal consistency, validity, and factor analysis were performed in this study. Independent *t*-test was used to compare between the two techniques of anesthesia, between boys and girls and among fathers and mothers.

**Results:**

Parental satisfaction mean values were higher in the computerized intraosseous anesthesia group in comparison to inferior alveolar nerve block (*P* value <0.05). The *T*-test showed that there was no difference between boys and girls regarding parental satisfaction (*P* value >0.05). Furthermore, fathers show lower satisfaction in the computerized interosseous anesthesia group (*P* value <0.05). Excellent internal consistency of this scale was resulted as Cronbach's alpha reliability coefficient was 0.985. After factor analysis, seven factor components were retained by using varimax rotation.

**Conclusions:**

Findings of this study reported that the designed parental satisfaction of dental local anesthetic techniques scale (PSLAS) is a valid and reliable scale to be used. Moreover, this study showed that parental satisfaction was higher when computerized intraosseous anesthesia was used in comparison to inferior alveolar nerve block.

## 1. Introduction

Dental local anesthesia (DLA) has an essential role in the success of dental procedures as it enables the practitioners to make immense therapeutic advances by controlling the pain caused [1]. However, DLA administration itself may also induce anxiety and pain due to several factors, including the unique characteristics of mucosal tissues, injection speed, and needle insertion depth [2–4].

Additionally, DLA is associated with systemic and local complications, including hematoma, edema, paresthesia, aphthous ulcers, facial paralysis, trismus, diplopia, soft tissue injuries, dose toxicity, allergy, and methemoglobinemia [5–8].

Consequently, the development of pain-free, noncomplicated local anesthetic techniques that induce patient relaxation during dental procedures is crucial for achieving patient satisfaction [9].

Patient satisfaction is a widely used index to measure the quality in healthcare, and analyzing patient satisfaction surveys has become a key method for obtaining patient feedback on local anesthetic techniques used in dentistry [10]. This can help in getting attention about the need to advance new local anesthetic delivery systems and techniques in the dental daily practice [11, 12]. However, most studies assessing patient satisfaction with dental local anesthesia have focused on adult patients and used questionnaires that have not been validated or assessed for their psychometric characteristics [12, 13].

In children, the ability to understand information related to the treatment options, techniques used, and associated complications is lacking and differs with age and maturity [14]. Thus, parents and legal guardians are most often the opportune alternate decision-makers for children [15]. According to several studies, children under the age of 16 are unable to make decision, provide consent, and express their logical satisfaction regarding dental procedures [16, 17].

Accordingly, assessment of parental satisfaction is a convenient way to reflect the child's acceptance of the regarded treatment. However, to date, there are no studies on parental satisfaction with dental local anesthetic techniques in their children and little is known about the associated factors.

Therefore, the aims of the present study were to study the validity and reliability of a novel scale that assess the satisfaction of parents/guardians with dental local anesthetic techniques for their children.

## 2. Materials and Methods

### 2.1. Study Design

This study was designed as a cross-sectional, observation study and conducted according to the COSMIN checklist over a period of 8 months from February 2022 to September 2022, and it was performed in accordance with the Declaration of Helsinki and was approved by the Research Ethics Committee of Faculty of Dentistry of Damascus University on 22-09-2021 (3662).

### 2.2. Study Participants and Groups

This study was conducted on 150 parents; each had one cooperative healthy child (definitely positive according to the Frankl behavior rating scale (FBRS)) [18], aged between 6 and 9 years old and needs bilateral pulpotomy for primary mandibular second molars in order to perform the study in a split mouth design. Children who have any systemic disease that makes them contraindicated to be anesthetized or children who have been referred to be sedated or generally anesthetized due to their uncooperativeness during the local anesthesia were excluded from this study.

After receiving demonstrations regarding the objectives and steps of the study, informed consent was signed from the parents who agreed to participate. Parents were fully informed about the nature of the study, any potential risks or benefits, and their right to withdraw from the study at any time. After performing the dental diagnosis to ascertain that the child matches the inclusion criteria, the first session was started with the selected type of DLA (according to the randomization). After 10 days, the second appointment was scheduled to perform the second type of DLA.

This study compared the parental satisfaction regarding two types of dental local anesthesia in children; the intervention type was computerized intraosseous anesthesia (QuickSleeper5) (QS) and the control one was an active control with an inferior alveolar nerve block (IANB). All participants were allocated equally into two groups: Group 1: starts the first session with QS and the second session with IANB and Group 2: starts the first session with IANB and the second session with QS. Therefore, a total of 300 questionnaires were analyzed as each parent filled out the questionnaire twice.

### 2.3. Randomization

In this study, the type of randomization used was simple randomization, which involves randomly assigning participants to different groups without any restrictions or stratifications based on factors such as age, sex, or disease severity.

The participants were randomly allocated to the two study groups using a computer-generated random allocation list obtained from the website https://www.randomalist.com. Each participant was assigned a number from 1 to 150 and then randomly allocated to one of the two groups. To determine which side of the mouth to start with, each child selected a sealed envelope that contained a card indicating the starting side (right or left).

### 2.4. Sample Size

The sample size was calculated according to the following formula that was conducted by Kothari [19]. As a result, the sample size was 277 parents and we have raised the number to set the sample size of this study as 300 parents.

(1) $$\begin{array}{c} n=\frac{P\left(1-P\right)}{A^{2}/Z^{2}+\left(P\left(1-P\right)/N\right)/R}\text{,} \end{array}$$

where *n* is the sample size. *N* is the population size, which was considered as the number of patients treated in the department of pediatric dentistry in Damascus University within the last three months (*N* = 3000). *P* is the estimated variance for the population, as it was assumed that almost 50% of the population would agree to the statements of the scale (*P*=0.5). *A* is the desired precision, which depends on the margin of the error that was set as 0.05. *z* was 1.96 as the confidence level of this study was 95%. *R* is the response rate of the audience, and it was set as 85%.

### 2.5. Instrument Development

A literature search was conducted using PubMed and Medline by inserting the following keywords: parental satisfaction, anesthesia, local anesthesia, questionnaire, scale, instrument, and validation. Several questionnaires have been developed and validated but none of them was specifically aimed to answer our topic. Based on the literature review, a draft scale comprising 18 items was developed.

After that, a team of voluntary professionals (one professor in pediatric dentistry, three PhD candidates in pediatric dentistry, one practitioner in pediatric dentistry, and one professor in psychology) was asked to help in improving the draft items. They used the brainstorming method to remove some items, improve ones, and add others, taking into consideration the requirements needed to serve the objective of the study [20]. They deleted two items, improved 10 items, and added 4 items. As a result, a 20-item scale with a 5-point Likert scale format was developed as a final draft. The answers were assigned as follows: 1: strongly disagree, 2: disagree, 3: not sure, 4: agree, and 5: strongly agree. A Likert scale was used to identify the level of agreement or disagreement with the statements. The items of the designed scale were created to be straightforward, easy to understand, and all related to the same variable in order to reduce the potential for misinterpretation of the results. All items were translated into the local Arabic language to be more understandable for the participants. In addition, about half of the items were written in a negative form. The scoring for these negative items was reversed in order to align with the direction of the positive items.

### 2.6. Testing the Scale

#### 2.6.1. Content Validity

Measuring content validity involves assessing individual items of a scale by asking experts if each item reaches the aim that the scale is designed to cover [21]. Six experts (three professors in pediatric dentistry and three professors in psychology) were asked to assess the items. In this study, the Lawshe formula [22] was used to determine the content validity ratio (CVR). For that reason, we asked each expert to determine whether the information behind each item is “essential,” “useful but not necessary” or “not necessary.”

(2) $$\begin{array}{c} CVR=\frac{N_{e}-\left(N/2\right)}{N/2}\text{,} \end{array}$$

where *N*_*e*_ is the number of essentials for the item. *N* is the number of experts.

#### 2.6.2. Criterion Validity

There are several types of criterion validity, and concurrent validity is one of them. Unlike other types of criterion validity, concurrent validity does not require a previous measure to correlate with the measure being studied [23].

In the present study, we assessed the concurrent validity of the satisfaction scale by calculating Pearson's correlation coefficient between the satisfaction scores obtained using the studied scale and the scores obtained using the FLACC (Face-Legs-Activity-Cry-Consolability) scale. If there were a high negative correlation between the FLACC scores and the parental satisfaction scores, it would indicate that when the child experiences higher levels of pain, the PSLAS is lower. This would suggest that the satisfaction scale is accurately measuring satisfaction with the local anesthetic technique used for the child.

#### 2.6.3. Construct Validity

Known-group validity was used in this study to assess the construct validity of the studied scale. For that reason, we divided the sample into two main groups:

  Group A: parental satisfaction was studied after their children have experienced inferior alveolar nerve block (IANB), *n* = 150.
  Group B: parental satisfaction was studied after the use of computerized intraosseous anesthesia using QuickSleeper 5 (Dental hi tech, France), *n* = 150.

As it is supposed according to many studies that QuickSleeper 5 causes less pain than IANB [24, 25], the results of the studied scale should express higher parental satisfaction in Group B than those in Group A in order to confirm the construct validity of this scale.

#### 2.6.4. Test-Retest Reliability

It involves administering the same measurement instrument to about 20% of the individuals under the same conditions after some period of time [26]. In this study, 20% of the total sample (30 participants) were randomly selected to refill the scale once again after one week. Those participants were taken from the completely selected sample.

Test-rest reliability was estimated with correlations between the scores at time point 1 and those at time point 2. Correlation coefficient (*r*) values were considered good if *r* ≥ 0.70 [27].

#### 2.6.5. Internal Consistency Reliability

Internal consistency concerns the extent to which items on the test or the instrument are measuring the same thing. This can be measured by Cronbach's alpha statistics [27].

Cronbach's alpha is computed by correlating the score for each scale item with the total score for each respondent [27].

### 2.7. Statistical Analysis

IBM SPSS software v. 25 was used to perform the statistical analysis. Descriptive statistics including calculation of mean and percentages of participated fathers and mothers and the percentages of children in relation to their gender were applied. The mean score for all participants and the sum of PSLAS were also measured. The text also mentions that group comparisons of parental satisfaction scores were conducted using independent *t*-tests to check for significant differences between IANB and QS, between boys and girls and between fathers and mothers (the level of significance (*P* value) and power of the study were set at 0.05% and 90%, respectively.

A factor analysis was also performed on the data, including steps such as determining the suitability of the data for factor analysis and extracting the number of components using principal component analysis. The retained components were rotated using varimax rotation, and seven factors with eigenvalues greater than 1 were retained. Factors with coefficients greater than 0.4 were used to interpret the suggested components, and the internal consistency of these factors was analyzed using the test of alpha Cronbach.

## 3. Results

The final sample was composed of 150 participants (102 mothers and 48 fathers), who were asked to fulfill the scale twice (once after using IANB and once after using QS). As a result, 300 scales were analyzed. Each scale was developed with 20 items, and the answers were arranged as follows: 1: strongly disagree, 2: disagree, 3: not sure, 4: agree, and 5: strongly agree. Consequently, parental satisfaction was classified into five categories: pretty low (0–20), low (21–40), moderate (41–60), high (61–80), and pretty high (81–100). The children involved within this study were 70 boys and 80 girls. The percentage, minimum score, maximum score, mean, and standard deviation of parental satisfaction in both groups are summarized in Table 1. Skewness is a measure of symmetry of data. Kurtosis is a measure of whether the data are heavy or light-tailed. The skewness and kurtosis of the PSLAS in the IANB group were 0.242 and −1.386, respectively, whereas the skewness and kurtosis of the PSLAS in the QS group were −2.117 and 6.02, respectively.

After getting the CVR of all items, it was concluded that there was no item under the value of 0.75 of the CVR. The content validity index CVI, which is the mean of the CVR for all items, was 0.9, and this means that the scale developed was with high content validity.

Table 2 presents the item mean scores, which ranged in the IANB group from 2.46 for item 2 “I think that the tools used to anesthetize my child's tooth was painful.” to 4.432 for item 4 “All my questions were answered clearly before performing the anesthesia.” In the QS group, the item mean scores ranged from 2.98 for item 5 “I was afraid of complications related to the anesthetic technique.” to 4.36 for item 2 “I think that the tools used to anesthetize my child's tooth was painful.”

**Figure 1:**
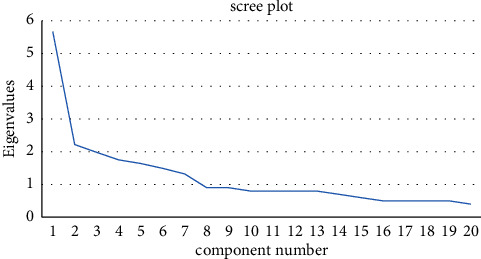
The eigenvalues scree plot.

The Kaiser–Meyer–Olkin (KMO) test of sampling adequacy gives a value of 0.84, and Bartlett's test of sphericity indicates the findings of 4533.376, d*f* = 123, and *P* value <0.001. These results indicate that our data are suitable for factor analysis.  Figure 1 represents the eigenvalues scree plot, and it shows that the items of the studied PSLAS can be presented by seven factors, which are the retained extracted components (eigenvalues >1.00). 

Initial Eigenvalues, percentages of variance, and cumulative percentages are summarized in Table 3. Factor 1 accounted for 6.33% of variance and is labeled as “Information” and composed of items (3 and 4). Factor 2 accounted for 6.69% of variance and is labeled as “Fear” and composed of items (1 and 14). Factor 3 accounted for 5.88% of variance and is labeled as “Cost” and composed of items (12 and 13). Factor 4 accounted for 12.33% of variance and is labeled as “Concern” and composed of items (5, 10, and 11). Factor 5 accounted for 31.11% of variance and is labeled as “Discomfort” and composed of items (2, 6, 7, 8, and 9). Factor 6 accounted for 20.11% of variance and is labeled as “Anesthesia-related sequelae” and composed of items (16, 17, 18, and 19). Factor 7 accounted for 6.77% of variance and is labeled as “Recommendation” and composed of items (15 and 20). All factors showed high internal consistency as Cronbach's alpha value was more than 0.5 for all of them. Varimax rotation results are represented in Table 4.

In order to study the criterion validity of the PSLAS, Pearson correlation showed that there was a strong negative correlation between the values of PSLAS and the values of FLACC with statistical significance (*P*=0.001 and Pearson correlation = −0.843). This means that the values of PSLAS have decreased when the pain levels of the children increased according to the FLACC scale. As a result, the studied PSLAS has a high criterion validity.

Furthermore, the independent sample *T*-test showed that there was a statistically significant difference between the values of PSLAS of the two groups (IANB and QS) with higher levels of parental satisfaction in the QS group. These findings indicated that the studied scale has high construct validity (known-group validity) as it can recognize the difference between the two techniques of anesthesia used in this study.  Table 5 shows the results of the *T*-test to study the difference between the two groups.

In addition, the independent *T*-test showed that there was no statistically significant difference between PSLAS mean values of the QS group despite of the sequence of the anesthetic technique (*t* value = 2.23 and *P* value <0.05). Moreover, there was no statistically significant difference between PSLAS mean values of the IANB group in relation to the sequence of the anesthetic technique that was used (*t* value = 2.03 and *P* value <0.05).

To study test-retest reliability, 20% of the participants (30 parents) have refilled one of their questionnaires two times. The correlation coefficient (*r*) of PSLAS scores between the two time points was 0.82. This means that the studied scale has a good reliability. In addition, Cronbach's alpha reliability coefficient was 0.985, which means that PSLAS has an excellent internal consistency.

Moreover, the Spearman–Brown Coefficient was 0.989 after dividing the scale into two parts (items with odd numbers and items with even numbers).

As it is presented in Table 6, there was no statistically significant difference in parental satisfaction between boys and girls in the two studied groups (IANB and QS).

On the other hand, there was a statistically significant difference in parental satisfaction between fathers and mothers in the two studied groups in the QS group only as fathers showed lower levels of satisfaction than mothers did.  Table 7 summarizes the results of the *t*-test.

## 4. Discussion

Parental satisfaction is a key aspect of the quality of care provided in pediatric dentistry [28]. DLA is one of the most important aspects to be studied due to its high impact on children's behavior management [29]. For that reason, DLA techniques are closely related to the level of parental satisfaction, as it can affect the child's future dental experiences and the parents' willingness to bring their child for regular dental check-ups [30]. Parental satisfaction with local anesthetic techniques is also linked to their trust in the dental team and their willingness to recommend the practice to others [31]. Correspondingly, PLSAS plays a crucial role in ensuring positive outcomes in pediatric dentistry and it is important for dental professionals to consider and evaluate parental satisfaction as a measure of the quality of care provided and to continuously strive to improve the techniques used to manage pain and anxiety in children [32]. As there is a lack of previous research on PLSA, we conducted a study to investigate this topic and developed a scale to gather data.

The developed scale consisted of 20 items and assigned a score between 21 and 100 in which higher scores indicated a better parental satisfaction towards the studied dental local anesthetic technique. It was intended to design an easy, affordable, and practical scale that can be used in both research and clinical settings. All efforts were made to write the questions in an easy language without incorporating medical terms in order to help parents in understanding the whole scale. The items of the designed scale were divided into two categories: (1) immediately after the procedure items, which involved 15 items and (2) on the day after items, which involved five items. This was done to allow parents to concentrate as they answered the first category directly after finishing the procedure, and they answered the second one on the day after the anesthesia as it was related to the anesthesia-related sequelae.

The studied PSLAS was designed as a Likert scale questionnaire in which each item should be answered with a value between 1 and 5 that ranges from strongly disagree to strongly agree. The Likert scale questionnaire is used to measure the intensity of an individual's agreement or disagreement with a statement [33]. It also provides a quantitative measure of attitudes, easy to administer and understand, and it can be used to measure a wide range of variables [33]. In addition, half of the developed items were formulated in a negative wording to increase the validity and reliability of the scale, to control for potential response biases, and to provide a more balanced assessment of the construct or variable being measured [34]. All questionnaires that had missing answers were excluded from the study.

Six experts were asked to review the first draft of the scale and to provide ratings of the relevance and importance of each item. Their ratings resulted in the CVR of 0.9, which means that the scale has high content validity. As well, concurrent validity was studied by analyzing the correlation between the FLACC scale and PSLAS. This validity is considered a type of criterion validity, and it was used in the present study, as there is no gold standard scale related to parental satisfaction with dental local anesthetic techniques [35]. Pain caused by local anesthesia can have a significant negative effect on parental satisfaction with the dental treatment provided [36]. In this regard, a high negative correlation between FLACC and PSLAS values was found in this study (−0.843) and this indicates that high scores on the FLACC scale are associated with low levels of parental satisfaction with the anesthesia, and vice versa.

Several studies showed that computerized intraosseous anesthesia causes less pain and complications than the traditional methods [37, 38]. Therefore, we divided our participants into two groups to find out if the designed scale can notice the difference in parental satisfaction between the groups (IANB and QS). This study revealed that higher levels of satisfaction resulted in the QS group, which means that PSLAS has high known-group validity. These findings can be attributed to the lower level of pain caused by computerized intraosseous anesthesia in comparison to IANB, which are in agreement with many previous studies [39, 40]. The mean value of satisfaction of each item in the studied scale was higher in the QS group than in the IANB group except for item 5 “I was afraid of complications related to the anesthetic technique.” This can be attributed to that intraosseous anesthesia is not a traditional routine method of anesthesia and little is known about its armamentarium, pain caused, effectiveness, and complications. As a result, more concern about the complications was shown in the QS group.

Moreover, factor analysis was used to estimate the validity of a parental satisfaction scale by identifying the underlying constructs or dimensions that the scale is measuring [41, 42]. After the factorial analysis, seven components of parental satisfaction were identified (information, fear, cost, concern, discomfort, anesthesia-related sequelae, and recommendation). However, due to the absence of prior studies in this area, it was challenging to compare our results with those of other investigations.

The results of this study have demonstrated that the developed PSLAS has excellent internal consistency. Cronbach's alpha coefficient for the scale was found to be 0.985, which is considered excellent. This high coefficient suggests that the items in the scale are measuring the same construct consistently and accurately, indicating that the scale is a reliable measure of parental satisfaction. These findings are particularly important as a reliable and consistent measure of parental satisfaction can enhance its usefulness in clinical practice and research. Therefore, the results of this study suggest that the developed scale can be a valuable tool to assess PSLA, and future research could further investigate its reliability and validity in different populations and settings.

The scale developed in this study was focused on Syrian parents in Damascus. Additional research is required to gather more scientific data that confirms the applicability of our scale to different groups of cultures and in different languages. It was difficult to calculate the time required by the respondents to fill all items of the scale, as each scale has two groups of items that will be filled in two different time points (immediately after the procedure items and on the day after items).

The current study showed that there was no significant difference in the values of PSLAS among boys and girls. This can be attributed to that many items of our scale are not related to the child specifically. Instead, tools, cost, complications, and information are some of the domains that are handled within our scale. However, the present study reported that fathers showed a lower level of satisfaction in the QS group than mothers did. This can be justified by the fact that fathers may have different communication styles or comfort levels when discussing healthcare decisions with healthcare providers [43]. They may not be as involved in the decision-making process for their child's dental care and may not have the same level of input or information about the procedure as mothers' findings [44]. Another explanation of those findings may be the higher cost of computerized intraosseous anesthesia which could be a factor that affects fathers' satisfaction with the procedure [45]. Dental care, like any other healthcare service, can be costly, and the cost of the procedure may be a concern for parents, especially in a context where the Syrian crisis has had a significant impact on the economy and the purchasing power of the population.

PSLAS is a valid and reliable tool to be used to assess the parental satisfaction with the dental local anesthetic technique. However, it needs to be translated into several languages in order to assess its validity and reliability before applying this scale in different countries in which other languages are spoken.

The present study has many strengths. It has developed a new tool to measure parental satisfaction, which can be useful for future research and clinical practice. In addition, the developed scale has been assessed for its validity and reliability, ensuring that the results obtained are accurate and trustworthy. The study also has practical implications for clinical practice by providing a tool that can help practitioners assess parental satisfaction with dental local anesthetic techniques and improve the quality of care provided to patients.

One of the limitations of our study is the lack of the external criterion, as there is no well-established gold standard scale that is designed to evaluate the same research problem. However, concurrent validity was assessed to counteract the absence of the criterion.

## 5. Conclusions

The present study is the first to design a parental satisfaction with the dental local anesthetic technique scale. Findings of this study reported that the designed PSLAS is a valid and reliable scale to be used. Moreover, this study showed that parental satisfaction was higher when computerized intraosseous anesthesia was used in comparison to inferior alveolar nerve block. Future research can explore the influence of different demographic characteristics of children and/or parents on the results of the scale. Furthermore, the designed scale should be translated into different languages to expand the population into several cultures.

## Figures and Tables

**Table 1:** Percentage, mean, min, and max of PSLAS in both groups.

PSLAS	Pretty low	Low	Moderate	High	Pretty high	Min	Max	Mean	SD^*∗*^
IANB group	1 (0.7%)	59 (39.3%)	20 (13.3%)	55 (36.7%)	15 (10%)	28	98	53.26	21.863
QS group	0 (0%)	4 (2.7%)	6 (4%)	48 (32%)	92 (61.3%)	29	100	82.11	12.904

^*∗*^Standard deviation.

**Table 2:** Item mean scores of both groups.

Item	Mean of values (IANB)	Mean of values (QS)
*Immediately after completing the procedure*		
(1) My child was not afraid of the tools used in local anesthesia	2.513	4.333
(2) I think that the tools used to anesthetize my child's tooth was painful	2.460	4.360
(3) The information that was provided to me about the technique of anesthesia was insufficient	4.112	4.260
(4) All my questions were answered clearly before performing the anesthesia	4.432	4.229
(5) I was afraid of complications related to the anesthetic technique	4.111	2.980
(6) I think that my child was feeling pain during the anesthesia	2.673	3.740
(7) My child had no pain during the dental procedure he received after the anesthesia	2.626	4.111
(8) My child was happy and smiley after finishing the appointment	2.773	4.009
(9) When I asked my child about his feeling during the anesthesia, he/she told me that it was bad	2.560	4.001
(10) I think that my child's experience with this anesthetic technique will encourage him/her to treat his/her teeth in the future	2.913	4.211
(11) I think that my child will be against coming to the dental clinic next time.	2.733	3.987
(12) I have no objection to pay extra expenses to comfort my child and to avoid hurting him/her	3.211	3.999
(13) I think that using this technique of anesthesia is considered as unnecessary welfare	3.875	4.001
(14) I think that this technique of anesthesia decreased my child's dental fear	2.555	4.230
(15) I will not repeat my child's experience once again for me or for my children	2.677	4.325

*One day after the anesthesia*		
(16) There was no lip biting to be reported after the anesthesia	3.011	4.310
(17) There was no ulcer that could complain my child after anesthesia	2.976	4.221
(18) Eating after the anesthesia was annoying to my child	2.987	4.122
(19) My child did complain some disturbance from the area of anesthesia	2.654	4.299
(20) I will not recommend others to use this technique of anesthesia for their children	2.677	4.289

**Table 3:** Findings of factor analysis.

Component	Initial eigenvalues		
	Total	% of variance	Cumulative %
1	5.67	6.33	6.33
2	2.22	6.69	13.02
3	1.98	5.88	18.9
4	1.73	12.33	31.23
5	1.64	31.11	62.34
6	1.49	20.11	82.45
7	1.32	6.77	89.22

**Table 4:** Results of varimax rotation.

Item	Components						
	1	2	3	4	5	6	7
*Information*							
3-The information that was provided to me about the technique of anesthesia was insufficient	0.883						
4-All my questions were answered clearly before performing the anesthesia	0.822						

*Fear*							
1-My child was not afraid of the tools used in local anesthesia		0.821					
14-I think that this technique of anesthesia decreased my child's dental fear		0.796					

*Cost*							
12-I have no objection to pay extra expenses to comfort my child and to avoid hurting him/her			0.711				
13-I think that using this technique of anesthesia is considered as unnecessary welfare			0.745				

*Concern*							
5-I was afraid of complications related to the anesthetic technique				0.822			
10-I think that my child's experience with this anesthetic technique will encourage him/her to treat his/her teeth in the future				0.744			
11-I think that my child will be against coming to the dental clinic next time				0.732			

*Discomfort*							
2-I think that the tools used to anesthetize my child's tooth was painful					0.852		
6-I think that my child was feeling pain during the anesthesia					0.875		
7-My child had no pain during the dental procedure he received after the anesthesia					0.833		
8-My child was happy and smiley after finishing the appointment					0.834		
9-When I asked my child about his feeling during the anesthesia, he/she told me that it was bad					0.864		

*Anesthesia-related sequelae*							
16-There was no lip biting to be reported after the anesthesia						0.773	
17-There was no ulcer that could complain my child after anesthesia						0.894	
18-Eating after the anesthesia was annoying to my child						0.799	
19-My child did complain some disturbance from the area of anesthesia						0.762	

*Recommendation*							
15-I will not repeat my child's experience once again for me or for my children							0.722
20-I will not recommend others to use this technique of anesthesia for their children							0.866

**Table 5:** Results of the independent *t*-test to study the difference between two groups.

Group	*N* ^*∗*^	Mean	SD	*t*-test	Degree of freedom	*P* value
IANB	150	53.26	21.863	13.919	298	0.001^*∗∗*^
QS	150	82.11	12.904			

^*∗*^The total number of questionnaires studied. ^*∗∗*^Statistical significance.

**Table 6:** Results of the *t*-test in studying the difference between boys and girls.

Group	Mean of PSLAS in boys	SD^*∗*^ of boys	Mean of PSLAS in girls	SD^*∗*^of girls	*T* value	*P* value
IANB	51.27	11.89	55.25	10.55	1.65	0.243
QS	80.17	13.88	84.08	12.89	1.98	0.255

^*∗*^Standard deviation.

**Table 7:** Results of the *t*-test in studying the difference between fathers and mothers.

Group	Mean of PSLAS in fathers	SD^*∗*^of fathers	Mean of PSLAS in mothers	SD^*∗*^of mothers	*T* value	*P* value
IANB	50.13	10.11	56.12	10.79	1.87	0.222
QS	75.12	11.19	89.01	10.14	3.75	0.002^*∗∗*^

^*∗*^Standard deviation. ^*∗∗*^Statistical significance.

## Data Availability

The data used to support the findings of this study are available from the corresponding author upon request.

## References

[1] Arapostathis K. N., Sixou J.-L. (2022). Local anesthesia in pediatric dentistry. *Pediatric Dentistry*.

[2] McPherson J. S., Dixon S. A., Townsend R., Vandewalle K. S. (2015). Effect of needle design on pain from dental local anesthetic injections. *Anesthesia Progress*.

[3] Nakai Y., Milgrom P., Mancl L., Coldwell S. E., Domoto P. K., Ramsay D. S. (2000). Effectiveness of local anesthesia in pediatric dental practice. *The Journal of the American Dental Association*.

[4] Mundiya J., Woodbine E. (2022). Updates on topical and local anesthesia agents. *Oral and Maxillofacial Surgery Clinics of North America*.

[5] Decloux D., Ouanounou A. (2021). Local anaesthesia in dentistry: a review. *International Dental Journal*.

[6] Aps J., Badr N. (2020). Narrative review: the evidence for neurotoxicity of dental local anesthetics. *Journal of Dental Anesthesia and Pain Medicine*.

[7] Cummings D. R., Yamashita D.-D. R., McAndrews J. P. (2011). Complications of local anesthesia used in oral and maxillofacial surgery. *Oral and Maxillofacial Surgery Clinics of North America*.

[8] Long B., Chavez S., Gottlieb M., Montrief T., Brady W. J. (2022). Local anesthetic systemic toxicity: a narrative review for emergency clinicians. *The American Journal of Emergency Medicine*.

[9] Smolarek P. D. C., Wambier L. M., Siqueira Silva L., Chibinski A. C. R. (2020). Does computerized anaesthesia reduce pain during local anaesthesia in paediatric patients for dental treatment? A systematic review and meta‐analysis. *International Journal of Paediatric Dentistry*.

[10] Burgener A. M. (2020). Enhancing communication to improve patient safety and to increase patient satisfaction. *Health Care Management*.

[11] Alshammari Y., Almuthhin M., Alarajah A., Barri G. M., Alshammari H., Alshahrani S. (2018). Patients satisfaction after endodontic treatment in Saudi Arabia. *The Egyptian Journal of Hospital Medicine*.

[12] Al-Obaida M. I., Haider M., Hashim R. (2019). Comparison of perceived pain and patients’ satisfaction with traditional local anesthesia and single tooth anesthesia: a randomized clinical trial. *World Journal of Clinical Cases*.

[13] Grace E. G., Barnes D. M., Reid B. C., Flores M., George D. L. (2003). Computerized local dental anesthetic systems: patient and dentist satisfaction. *Journal of Dentistry*.

[14] Hein I. M., De Vries M. C., Troost P. W., Meynen G., Van Goudoever J. B., Lindauer R. J. L. (2015). Informed consent instead of assent is appropriate in children from the age of twelve: policy implications of new findings on children’s competence to consent to clinical research. *BMC Medical Ethics*.

[15] Musmade P., Nijhawan L., Udupa N. (2013). Informed consent: issues and challenges. *Journal of Advanced Pharmaceutical Technology and Research*.

[16] Adewumi A., Hector M. P., King J. M. (2001). Children and informed consent: a study of children’s perceptions and involvement in consent to dental treatment. *British Dental Journal*.

[17] Coyne I., Amory A., Kiernan G., Gibson F. (2014). Children’s participation in shared decision-making: children, adolescents, parents and healthcare professionals’ perspectives and experiences. *European Journal of Oncology Nursing*.

[18] Frankl S. N., Shiere F. R., Fogels H. R. (1962). Should the parent remain with the child in the dental operatory?. *Journal of Dentistry for Children*.

[19] Kothari C. R. (2004). Sample size determination. *Research Methodology*.

[20] Omidvari A., Abedianpour S. (2018). Brainstorming strategy and writing performance: effects and attitudes. *Journal of Language Teaching and Research*.

[21] Almanasreh E., Moles R., Chen T. F. (2019). Evaluation of methods used for estimating content validity. *Research in Social and Administrative Pharmacy*.

[22] Ayre C., Scally A. J. (2014). Critical values for Lawshe’s content validity ratio: revisiting the original methods of calculation. *Measurement and Evaluation in Counseling and Development*.

[23] Boparai J. K., Singh S., Kathuria P. (2019). How to design and validate a questionnaire: a guide. *Current Clinical Pharmacology*.

[24] Beneito-Brotons R., Peñarrocha-Oltra D., Ata-Ali J., Peñarrocha M. (2012). Intraosseous anesthesia with solution injection controlled by a computerized system versus conventional oral anesthesia: a preliminary study. *Medicina Oral, Patología Oral y Cirugía Bucal*.

[25] Smaïl‐Faugeron V., Muller‐Bolla M., Sixou J., Courson F. (2019). Evaluation of intraosseous computerized injection system (QuickSleeper^TM^) vs conventional infiltration anaesthesia in paediatric oral health care: a multicentre, single‐blind, combined split‐mouth and parallel‐arm randomized controlled trial. *International Journal of Paediatric Dentistry*.

[26] Yaddanapudi S., Yaddanapudi L. N. (2019). How to design a questionnaire. *Indian Journal of Anaesthesia*.

[27] Fosgerau M., Karlström A. (2010). The value of reliability. *Transportation Research Part B: Methodological*.

[28] Tellez M., Kaur S. (2013). Caregivers’ satisfaction with pediatric dental care in a university clinical setting in North Philadelphia. *Journal of Dental Education*.

[29] Gupta A., Marya C. M., Bhatia H. P., Dahiya V. (2014). Behaviour management of an anxious child. *Stomatologiia*.

[30] Vellingiri S. (2015). Assessment of parent’s preference to general or local anesthesia for children undergoing dental treatment. *World Journal of Dentistry*.

[31] Yamamoto L. G., Young L. L., Roberts J. L. (1997). Informed consent and parental choice of anesthesia and sedation for the repair of small lacerations in children. *The American Journal of Emergency Medicine*.

[32] Halfon N., Inkelas M., Mistry R., Olson L. M. (2004). Satisfaction with health care for young children. *Pediatrics*.

[33] Joshi A., Kale S., Chandel S., Pal D. K. (2015). Likert scale: explored and explained. *British Journal of Applied Science and Technology*.

[34] Rattray J., Jones M. C. (2007). Essential elements of questionnaire design and development. *Journal of Clinical Nursing*.

[35] Webber T. A., Critchfield E. A., Soble J. R. (2020). Convergent, discriminant, and concurrent validity of nonmemory-based performance validity tests. *Assessment*.

[36] Franck L. S., Cox S., Allen A., Winter I. (2004). Parental concern and distress about infant pain. *Archives of Disease in Childhood - Fetal and Neonatal Edition*.

[37] Sixou J., Marie‐Cousin A., Huet A., Hingant B., Robert J. (2009). Pain assessment by children and adolescents during intraosseous anaesthesia using a computerized system (QuickSleeper^TM^). *International Journal of Paediatric Dentistry*.

[38] Smail-Faugeron V., Muller-Bolla M., Sixou J.-L., Courson F. (2015). Split-mouth and parallel-arm trials to compare pain with intraosseous anaesthesia delivered by the computerised Quicksleeper system and conventional infiltration anaesthesia in paediatric oral healthcare: protocol for a randomised controlled trial. *BMJ Open*.

[39] V Serikova O. V. (2013). Results of automated syringe “quicksleeper” intraosseous anesthesia in the therapeutic dentistry. *Medical Science Bulletin Center Chernozemye (Naučno-medicinskij Vestn. Cent. Černozemʹâ)*.

[40] Singh N., Wyzga S., Yune J., Mathur G., Błochowiak K. (2022). Comparison of conventional syringe anesthesia and three computer-aided anesthesia systems (Quicksleeper, SleeperOne, and the Wand). *European Journal of Clinical and Experimental Medicine*.

[41] Dashash M., Boubou M. (2021). Measurement of empathy among health professionals during Syrian crisis using the Syrian empathy scale. *BMC Medical Education*.

[42] Marsh H. W., Guo J., Dicke T., Parker P. D., Craven R. G. (2020). Confirmatory factor analysis (CFA), exploratory structural equation modeling (ESEM), and set-ESEM: optimal balance between goodness of fit and parsimony. *Multivariate Behavioral Research*.

[43] Vusio F., Thompson A., Birchwood M., Clarke L. (2020). Experiences and satisfaction of children, young people and their parents with alternative mental health models to inpatient settings: a systematic review. *European Child and Adolescent Psychiatry*.

[44] Sigurdardottir A. O., Garwick A. W., Svavarsdottir E. K. (2017). The importance of family support in pediatrics and its impact on healthcare satisfaction. *Scandinavian Journal of Caring Sciences*.

[45] Ekert-Jaffé O. (2011). Are the real time costs of children equally shared by mothers and fathers?. *Social Indicators Research*.

